# Development and evaluation of a speech-generating AAC mobile app for minimally verbal children with autism spectrum disorder in Mainland China

**DOI:** 10.1186/s13229-017-0165-5

**Published:** 2017-10-03

**Authors:** Sainan An, Xiaoping Feng, Yue Dai, Hongli Bo, Xiuqing Wang, Mu Li, John Zhuohao Woo, Xingmei Liang, Cheng Guo, Charles Xingchao Liu, Liping Wei

**Affiliations:** 1 School of Life Sciences, Beijing Normal University, No. 19, XinJieKouWai St., HaiDian District, Beijing, 100875 China; 2 National Institute of Biological Sciences, No. 7, Science Park Road, ZhongGuanCun Life Science Park, ChangPing District, Beijing, 102206 China; 3 AppChina, Bejing, China; 4 Beijing Stars and Rain Education Institute, No. 18, ShuangQiao East Road, Beijing, 100121 China; 5 Academy of Arts and Design, Tsinghua University, No. 1, QingHuaYuan Road, HaiDai District, Beijing, 100084 China; 6 Inway Design, Beijing, China; 7 G-Wearables, Inc., Beijing, China; 8 Center for Bioinformatics, State Key Laboratory of Protein and Plant Gene Research, School of Life Sciences, Peking University, No. 5, YiHeYuan Road, HaiDian District, Beijing, 100871 China

**Keywords:** Augmentative and alternative communication, App, Development, Training effectiveness, Mainland China

## Abstract

**Background:**

Mobile touchscreen devices are currently being used as speech-generating devices (SGDs) and have been shown to promote the communication skills, particularly the requesting skills of children with autism spectrum disorders (ASD) who have limited spoken language. However, no augmentative and alternative communication (AAC) mobile app has been developed and evaluated in the Chinese language in Mainland China.

**Methods:**

We developed an AAC mobile app, which is the first in Mainland China, to our knowledge, named Yuudee (Chinese name 小雨滴 (xiaoyudi)). Yuudee was developed using the Objective-C and Java programming languages. A five-phase training protocol for making requests using Yuudee was developed based on the Picture Exchange Communication System. We trained ten minimally verbal children with ASD to make requests using Yuudee and evaluated the effectiveness of the training.

**Results:**

Yuudee has a built-in library of over 400 pictures with corresponding spoken phrases that are divided into 39 categories ranging from making simple requests to expressing emotions. An additional important feature of Yuudee is its customization functions that allow a parent or trainer to easily select pictures and phrases to display, create new pictures and phrases, and change the layouts and orders of the pictures to fit the personal needs of each child. Yuudee is freely available in an iOS version from the iTunes App Store (https://itunes.apple.com/cn/app/xiao-yu-di/id794832934?mt=8) and in an Android version from Google Play (https://play.google.com/store/apps/details?id=com.supersuperstar.yuudee.vue) and domestic Chinese Android App stores. Three consecutive unprompted successful responses, which were defined as an initial training success, were achieved in at least three of the five phases for all ten of the evaluated children. The accuracy rate of a given phase was calculated for each child who achieved three consecutive unprompted successful responses in the phase. Seven children achieved at least 50% accuracy in at least two of the five phases. The other three children achieved at least 50% accuracy in only one phase. Two children achieved at least 50% accuracy in all of the phases in which they were trained.

**Conclusions:**

Our data suggest that Yuudee is a useful tool for helping minimally verbal children with ASD make requests.

**Electronic supplementary material:**

The online version of this article (10.1186/s13229-017-0165-5) contains supplementary material, which is available to authorized users.

## Background

Autism spectrum disorder (ASD) is a complex neurodevelopmental disorder characterized by deficits in social communication and social interaction and by restricted, repetitive patterns of behavior, interests, or activities [1]. The spoken language abilities of children with ASD are variable and range from high-level speech to the absence of spoken language. Approximately 30% of children with ASD are minimally verbal [2, 3]. They use a small repertoire of spoken words or fixed phrases or use stereotyped or scripted spoken language in a noncommunicative manner [4, 5].

Augmentative and alternative communication (AAC) systems, including gestures, sign language, the Picture Exchange Communication System (PECS), and speech-generating devices (SGDs), have been developed to improve the communicative capacity of children with limited functional spoken language. PECS and SGDs are commonly used for children with ASD. PECS is a visual-based system that aims to teach children how to communicate with others by exchanging pictures [6, 7]. PECS includes six phases that target communication skills ranging from making simple requests to making comments. Each phase of PECS is taught systematically using specific strategies and procedures that were developed based on the principles of applied behavior analysis [8]. Studies have demonstrated that PECS training can promote functional communication skills (e.g., requesting and commenting skills) and social communication skills in children with ASD [9–15]. Children with ASD who were trained to use PECS showed improvements in their verbal skills in some studies [14, 16]. In a randomized controlled study, PECS was more effective than Responsive Education and Prelinguistic Milieu Teaching for increasing the number of nonimitative spoken communication acts and the number of different nonimitative words used in the posttreatment period [17], although these differences were not found in a later follow-up period.

SGDs generate recorded or synthesized speech outputs when displayed pictures are activated by a user. Using SGDs can promote the communication skills of children with ASD, particularly their requesting skills [13, 18–20]. A review of 23 studies found that 78% of these studies provided conclusive evidence that using SGDs could improve the communication skills of children with ASD, although it should be noted that most of the studies targeted requesting skills only [21]. Using SGDs has been demonstrated to promote the functional speech of children with ASD in a study that used a blended, adaptive treatment design and targeted minimally verbal children aged 5–8 years [22]. Children who received SGDs at the beginning of the adaptive intervention gained significantly more spontaneous communicative language than children who received SGDs later or did not receive SGDs at all [22, 23].

In recent years, mobile touchscreen devices (e.g., iPads, iPhones, and Android phones) are increasingly being used as SGDs. Many AAC Apps (e.g., Proloquo2Go) that run on these mobile touchscreen devices have been developed [24, 25]. SGDs that consist of mobile touchscreen devices and AAC Apps have potential advantages such as the capacity to include many pictures, their portability, and their relatively low cost [26]. Studies have shown that using mobile devices with AAC Apps can improve the abilities of children with ASD to make single- or multiple-step requests [27–30] and to give social communication responses (e.g., “thank you”) after making requests [31]. Mobile touchscreen devices with AAC Apps are emerging as promising alternatives of traditional SGDs and PECS. Several studies have adapted and modified the detailed and empirically validated training procedures of PECS for use in SGD training with mobile devices [29, 32].

Sun et al. [33] systematically reviewed the literature regarding estimates of the prevalence of ASD or childhood autism in Mainland China, Hong Kong, and Taiwan. Their meta-analysis concluded that the prevalence of ASD in three areas was 26.6/10,000, which is approximately an order of magnitude lower than the estimated prevalence of 224/10,000 in the USA [34]. It should be noted that there is a lack of large-scale epidemiological investigations on ASD in Mainland China that use comparable methods to those used in studies from other developed countries. The percentage of minimally verbal children with ASD in Mainland China has not been reported. We are currently conducting a project called the Autism Genetic Research Project in Mainland China (presented in detail in the Section 2). Although this project is conducted at autism intervention centers instead of at the population level, it may still shed light on the percentage of minimally verbal children in Mainland China. Among 4–6-year-old children with ASD who participated in this project (sample size around 550), 50% were minimally verbal according to the definition by Thurm et al. [35]. Extrapolating this based on the aforementioned estimate of Sun et al., one could assume that there may be about 50,000 minimally verbal children with ASD aged 4–6 years in Mainland China.

No AAC Apps for minimally verbal children with ASD have been developed and evaluated in Mainland China. Considering the potentially large number of minimally verbal children with ASD in Mainland China and given the demonstrated benefits of AAC Apps in improving communication skills, we developed a freely available AAC mobile App named Yuudee that features a large built-in picture library and a set of customization functions. We evaluated the effectiveness of training for making requests using Yuudee in ten minimally verbal children with ASD. The phases of Yuudee training were designed based on both the training phases of PECS and the functionalities of Yuudee. Our analysis revealed that the use of Yuudee with our PECS-derived training procedures was effective in improving the requesting skills of minimally verbal children with ASD.

## Methods

### Development of Yuudee

Considering the likely overlap of certain basic communication needs across minimally verbal children with ASD, a built-in picture library was developed. In addition, Yuudee was designed to be easily customized. The pictures displayed for the child can be edited according to the specific communication needs of the child. The characteristics of children with ASD were considered during the development of Yuudee; for example, given that fine motor impairments are common in children with ASD [36], the number of pictures displayed on the screen can be changed from one to nine according to the fine motor skills of a given child.

To make Yuudee easily available to more families, both an iOS version and an Android version were developed. The iOS version of Yuudee was developed using the Objective-C programming language. The Android version of Yuudee was developed using the Java programming language. The relationships between the pictures, texts, voices, and category information were specified. When installing Yuudee, pictures and voices included in the built-in picture library are copied into the device. The coordinates of the pictures on the screen are calculated according to the setting for the number of pictures on the screen. In the iOS version of Yuudee, pictures included within a particular courseware or a given category are arranged in a horizontally oriented UIScrollView. Users can navigate between pages by scrolling the UIScrollView. In the Android version of Yuudee, the horizontal paging is fulfilled by customizing the ScrollLayout control.

In the iOS version of Yuudee, the AVFoundation framework is used for picture selection and voice recording when inputting a new picture. The UIImagepickerController is used to select pictures from the photo library or to take new photos using the built-in digital camera. In the Android version of Yuudee, the framework for creating new pictures was developed using self-developed tools. In both versions, the touch events on the pictures are captured. When a touch event is detected, the dynamic effect of the picture is played using UIView animation (the iPad Version) or the Android Animation application programming interface (the Android version) according to the setting of the picture. Three 100*100 pixel regions at the upper left, upper right, and bottom right corners of the screen are monitored for touch events. When touch events simultaneously occur in all of these three regions, Yuudee enters the edit mode where pictures that are accessible to children can be edited. The position of the pictures can be changed after a ‘long touch’ event on a picture is detected.

### Participants

Ten 3–6-year-old children who met our inclusion criteria were included in the present study. The inclusion criteria were (1) exceeding the cutoff for ASD of the Autism Diagnostic Observation Schedule (ADOS) and Autism Diagnostic Interview-Revised (ADI-R) [37, 38], which were conducted by certified practitioners; (2) having been diagnosed with ASD based on criteria in the Diagnostic and Statistical Manual of Mental Disorders, Fourth Edition-Text Revision by experienced child psychiatrists; (3) ‘minimally verbal’ based on item A1 ‘Overall level of non-echoed spoken language’ of the ADOS (Children whose A1 codes indicated no speech, single words, or occasional phrases were identified as minimally verbal children [35]); (4) having not been diagnosed with or suspected of having epilepsy; and (5) having never previously used any other speech-generating devices or PECS. All ten of the children were diagnosed as having autism by child psychiatrists and were assessed by the ADOS module 1. The Vineland Adaptive Behavior Scales (Chinese version) were used to evaluate the everyday personal and social skills of these children [39]. Descriptive characteristics of each child are presented in Table 1; age equivalent scores and standard scores on the Vineland Adaptive Behavior Scales (Chinese version) are listed in this table.

**Table 1:**
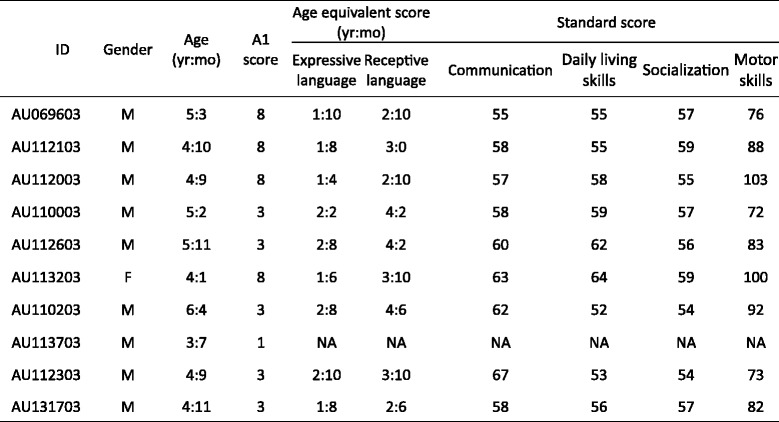
Descriptive characteristics of each minimally verbal child with ASD

A1 scores were from the Autism Diagnostic Observation Schedule

*NA* not available, *M* male, *F* Female

Because all the enrolled children also participated in our Autism Genetic Research Project, the identifiers used in the present study were the same as those in that project. The ongoing Autism Genetic Research Project is collecting phenotypic data and DNA samples from children with ASD and their parents to study the genetics of ASD in China. The families affected by ASD were recruited from intervention centers.

### Preferred reinforcers

Parents were asked to list the items and activities that were particularly favorable to their child before Yuudee training. The identification of individualized reinforcers is important to keep children motivated during the training. Pictures with recorded speech corresponding to the items and/or activities on each personalized list were generated before Yuudee training if no such pictures were present in the built-in library. The parent-selected reinforcers varied greatly across the children. Only a few reinforcers (e.g., biscuits and candies) were common among these children. For most of the reinforcers, digital photos taken of real objects were used during training as only a few common reinforcers were represented by pictures in the built-in picture library.

### Training design

The training for making requests with Yuudee was conducted in a classroom. Yuudee training consisted of eight 30-min sessions. One or two sessions were conducted per week for 5 weeks. iPad mini or iPad devices running Yuudee were used in this study. Each child was trained by two trainers (a communication partner and a physical prompter). Training was divided into five phases based on the phases of PECS and the functionalities of Yuudee (presented in detail below). The training procedures of each phase were adapted from the training procedures of PECS.

### Reinforcer assessment

At the beginning of each session, two potential reinforcers were put on the table between the child and the communication partner. The one chosen by the child was then used as the reinforcer. In the middle of each session, the procedure for choosing a reinforcer from two options was repeated to determine whether the reinforcer used in the remaining time of the session should be changed. Based on these choices, we assumed that children were motivated to obtain the preferred objects during training.

### Training phases and procedures

Phase I consisted of making requests by touching a single picture on the screen; this corresponds to the first phase of PECS. This phase aimed to help children to establish the ability to initiate a request by touching a picture on the screen. At the beginning of this phase, the communication partner showed the reinforcer to the child to gain his/her attention. If the child reached toward the reinforcer, the physical prompter immediately disrupted his/her reach by picking up the child’s right hand and then physically directing the child to touch the picture on the screen. When the recorded speech was broadcast by the device, the communication partner immediately spoke the name of the reinforcer and gave the reinforcer to the child. If the child did not initiate the request, full physical prompts were provided to help the child to touch the single picture on the screen. The number of full physical prompts offered was gradually reduced throughout the course of a single training phase.

Phase II consisted of discriminating between the pictures present on a single screen page; this phase corresponds to the third phase of PECS. Phase II of this study differs from the third phase of PECS in two ways: (1) the child and the communication partner continued to sit across a table, and (2) the discrimination was only made between highly preferred and non-preferred/unrelated items. This phase started by displaying two pictures on the screen. The communication partner showed both items to the child. If the child spontaneously touched one picture, the communication partner gave the corresponding item to the child. Prompts were provided to help the child to touch the target picture on the screen if the child did not initiate the request. When the child was able to select the target picture from two pictures, the number of pictures on a single screen was gradually increased. At most, nine pictures were displayed on a single screen page. Note that during this phase, the position of the target picture was changed frequently to avoid a situation where the child simply touched the same region of the screen repeatedly.

Phase III involved switching between multiple screen pages. Many pictures can be displayed by distributing them across multiple screen pages. In this phase, the pictures were put on two or more screen pages. An exemplary trial with full physical prompts was conducted to show the child how to switch between the screen pages. At the beginning of each trial, the screen page with the target picture was not the one displayed to the child. If the child did not spontaneously initiate the request, full physical prompts were provided to help the child to switch between different screen pages, to find the page with the target picture, and to touch the target picture. Prompts were gradually faded during this phase.

Phase IV corresponds to the second phase of PECS, with the aim of increasing the child’s spontaneity. This phase was further divided into phases IVA and IVB. In phase IVA, the distance between the child and the communication partner was gradually increased (eventually to as far as across the room). In phase IVB, there were distances between both the child and the communication partner and the child and the device. In both IVA and IVB, the communication partner moved around the room. To acquire the desired item, the child was required to travel to the communication partner and to touch the target picture in front of the communication partner. During phase IVB, the position of the iPad was frequently changed to enhance their consciousness of being required to initially go to take the iPad.

Phase V aimed to enable children to independently open the App. In this phase, the Yuudee icon was displayed on the screen. To enter Yuudee, the child needed to touch the Yuudee icon. In an exemplary trial, the physical prompter held the right hand of the child, guided the child to touch the Yuudee icon, and then guided the child to touch the target picture. In the following trials, full physical prompts were provided if the child did not initiate the request or touched the icon for another application.

Note that the second training phase that the children experienced was phase IV. Phase IV was typically conducted as a part of every session for each child but only after the training of the other phases (i.e., after each of phase I, phase II, phase III, and phase V). Phase III (switching between multiple screen pages) is an extension of phase II (picture discrimination) and allows children to select preferred items from an increasing number of options. Therefore, phase III occurred just after phase II.

During phases I, II, III, and V, the communication partner and the child sat across a table. The iPad mini or iPad was placed on the table between them. The physical prompter sat behind the child to provide physical assistance. As in PECS training, no verbal prompts were used during the training for making requests with Yuudee. During phases I and II, the reinforcers were always in view. During other phases, reinforcers could sometimes be placed below the level of the table and outside the children’s view. At the beginning of phase I, reinforcers were presented to the children by the communication partners for several (3–5) trials. During phase IV, the communication partners interacted with the reinforcers to get children’s attention when the children were not actively engaged in the training process. For example, the communication partners ate a preferred food or played with a preferred toy.

### Evaluation of training effectiveness

We recorded whether prompts were provided, which types of prompts were provided, and whether the child touched the target picture in each trial. An unprompted successful response was defined as spontaneously initiating a request and successfully touching the target picture without prompts to obtain the favored item in a trial. Using phase IVB as an example, an unprompted successful response was recorded if a child spontaneously traveled to take the iPad and then traveled to the trainer and touched the picture that corresponded to the reinforcer in front of the trainer without using prompts.

The training effectiveness of a given phase was measured in two steps. We initially evaluated whether the child achieved three consecutive unprompted successful responses in this phase. The achievement of three consecutive unprompted successful responses was regarded as the beginning of acquiring competence for a given phase. If a child achieved three consecutive unprompted successful responses in a given phase, then we calculated the accuracy rate (the proportion of unprompted successful responses) among the trials conducted after this initial achievement of three consecutive unprompted successful responses in this phase. In this study, we considered a threshold of at least 50% accuracy as ‘accurate’ because the training consisted of only eight 30-min sessions and five training phases were evaluated.

### Inter-observer agreement

To assess the reliability of the data collection, two independent observers collected performance data on all sessions for all ten of the children. The inter-observer agreement (IOA) for each session was calculated. The IOA ranged from 94.28 to 100%, and the mean IOA was 98.81%.

### Procedural integrity

To assess whether the procedures were correctly implemented in the proper sequence, training procedure checklists were created for each phase (Additional file 1: Table S1). Data on procedural integrity were collected for 50% of the sessions of each child. The procedural integrity values ranged from 91.34 to 100% with a mean of 99.20%.

## Results

### Features of Yuudee

**Fig. 1:**
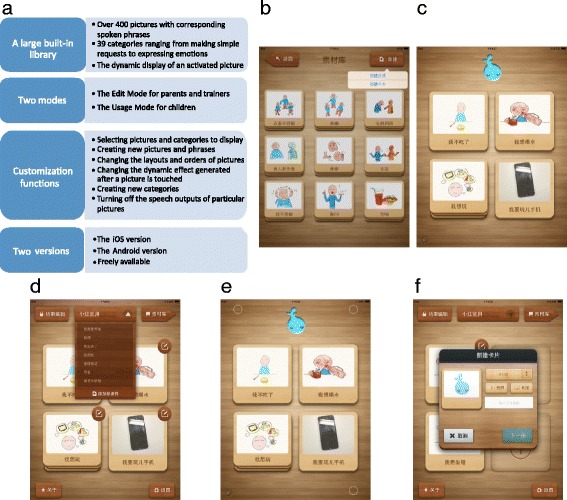
Yuudee features. **a** Summary of Yuudee features. **b** The built-in picture library. **c** The Usage Mode of Yuudee. Pictures or categories that are selected from the built-in library or are made by parents or trainers according to the specific communication needs of the child are displayed on the screen. **d** The Edit Mode of Yuudee. In the Edit Mode, parents or trainers can edit the content that is accessible to the child using the customization functions of Yuudee. **e** The three-point touch gesture used to enter the Edit Mode. **f** The interface used for inputting a new picture

The built-in library of Yuudee includes over 400 line drawings. Each picture is associated with corresponding text (usually a Chinese phrase) that was recorded by a Mandarin-speaking boy. These pictures have been organized into 39 categories, including categories about ‘communication functions’ ranging from making simple requests to expressing emotions, categories about cognition (e.g., colors, numbers, and shapes), and a category containing social stories (Fig. 1b). When a picture from a communication function category is touched, three pictures that represent three consecutive actions are displayed in order with a short time interval between each picture. Therefore, upon the selection of a picture, Yuudee presents an attractive dynamic display of the user-selected picture; this differs from the static displays that are typical of many other currently available AAC Apps.

Yuudee has two modes: the Usage Mode for children (Fig. 1c) and the Edit Mode for parents and trainers (Fig. 1d). The default mode is the Usage Mode when opening Yuudee. In the Usage Mode, pictures and categories selected and edited by parents or trainers are displayed on the screen. To enter the Edit Mode, parents or trainers need to simultaneously touch three points on the upper left, upper right, and bottom right corners of the screen (Fig. 1e); this was designed to prevent children from inadvertently entering the Edit Mode.

Yuudee can be flexibly customized to suit the personal communication needs of the child as well as the particular operator level at which the child can effectively use Yuudee. Parents or trainers initially edit the content to be displayed to the child by either selecting an existing courseware unit or creating a new courseware unit. In a particular courseware unit, parents or trainers can add and delete pictures or categories. Pictures can be added by selecting built-in pictures or by creating new pictures using three steps: (1) selecting digital photos from the photo library or taking new photos using the built-in digital camera, (2) inputting text, and (3) recording speech (Fig. 1f). The number of pictures displayed on the screen can be changed from 1 to 9 using the layout setting of the pictures, thereby accommodating the fine motor skills of the child and allowing expanded options as the training phases progress. There are three types of layouts: “1 × 1,” “2 × 2,” and “3 × 3.” The orders of the pictures displayed on the screen can be changed; this makes it possible, for example, to test whether the child is able to choose the right picture wherever it is on the screen during training. Parents or trainers can set the dynamic effect that is generated after a picture is touched; there are two types of dynamic effects: “Magnification” and “Magnification and rotation.” New categories can be added to the picture library. The speech output that is generated after a picture is touched can be turned off.

There are two versions of Yuudee: one for Apple iOS and another for the Android platform. Parents can find Yuudee by searching for its Chinese name “xiaoyudi” or “小雨滴” in the iTunes App Store or in Google Play and domestic Chinese Android App stores. They can then freely install Yuudee. Yuudee runs on iPads and Android phones. Parents can choose between the two versions of Yuudee according to the touchscreen devices they have.

### Download statistics of Yuudee

The iOS version of Yuudee was released in April 2014. According to the data from April 2015 to November 2016, the iOS version of Yuudee was downloaded 1995 times from the iTunes App Store with an average of approximately 100 downloads per month. The Android version of Yuudee was released in April 2015. By November 2016, the Android version of Yuudee was downloaded 35,000 times from Google play and another eight domestic Android App stores. Thus, Yuudee was downloaded more than 37,000 times by November 2016.

### Evaluation of Yuudee training

**Fig. 2:**
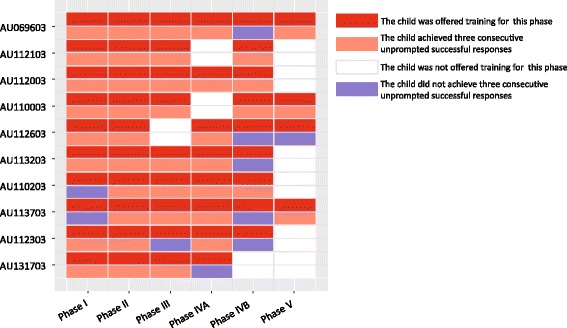
The status of the achievement of three consecutive unprompted successful responses in each phase for each child. Ten minimally verbal children with ASD participated in the training for making requests using Yuudee

For each training phase (I–V), we checked whether children were able to achieve three consecutive unprompted successful responses. In phase I, eight of the ten trained children successfully made three consecutive unprompted successful requests after an average of 36.38 trials (Fig. 2). In phase II, all ten of the trained children achieved three consecutive unprompted successful responses after an average of 9.2 trials (Fig. 2). In phase III, eight of the nine trained children made three consecutive unprompted successful requests after an average of 45.5 trials (Fig. 2). A total of 44 trials were conducted with AU112303 who failed to achieve three consecutive unprompted successful responses in phase III. In phase IVA, seven of the eight trained children made three consecutive unprompted successful requests after an average of 33.71 trials (Fig. 2). A total of 41 trials were conducted with AU131703 who failed to achieve three consecutive unprompted successful responses in phase IVA. In phase IVB, four of the nine trained children achieved three consecutive unprompted successful responses after an average of 15.5 trials (Fig. 2). A total of 19–65 trials were conducted with the five children who failed to achieve three consecutive unprompted successful responses in phase IVB. The numbers of children trained in phase IVA and phase IVB were different because some trainers skipped phase IVA and directly conducted phase IVB. Among the four children who were trained in phase V, three of them made three consecutive unprompted successful requests after an average of 4.33 trials (Fig. 2). A total of 21 trials were conducted with AU112603 who failed to achieve three consecutive unprompted successful responses in phase V. It is noteworthy that the number of trials conducted before the achievement of three consecutive unprompted successful responses was highly variable among these children. For example, while 3 trials were conducted before AU113203 achieved three consecutive unprompted successful responses in phase I, a total of 78 trials were conducted before AU112103 achieved this same level.

All ten of the children achieved three consecutive unprompted successful responses in at least three of the five phases (Fig. 2). Three children (AU112103, AU112003, and AU110003) achieved three consecutive unprompted successful responses in all of the phases in which they were trained. Two children (AU069603 and AU113203) made three consecutive unprompted successful requests in all of the phases in which they were trained with the exception of phase IVB. AU112603, AU110203, AU112303, and AU131703 achieved three consecutive unprompted successful responses in three of the five phases. AU110203 and AU113703 did not achieve three consecutive unprompted successful responses in phase I. As they successfully made three consecutive unprompted successful requests in the subsequent phases, we assume that they actually had the ability to achieve this in phase I. We assume that their failure in phase I was caused by the minimal number of trials (3 or 5) that the trainers conducted with them in phase I.

Table 2 lists the accuracy rate after three consecutive unprompted successful responses were achieved in each phase for each child. Six of the eight children who achieved three consecutive unprompted successful responses in phase I achieved at least 50% accuracy in phase I (Table 2). Eight of the ten children who achieved three consecutive unprompted successful responses in phase II achieved at least 50% accuracy in phase II (Table 2). Four of the eight children who achieved three consecutive unprompted successful responses in phase III achieved at least 50% accuracy in phase III (Table 2). Four of the seven children who achieved three consecutive unprompted successful responses in phase IVA achieved at least 50% accuracy in phase IVA (Table 2). Three of the four children who achieved three consecutive unprompted successful responses in phase IVB achieved at least 50% accuracy in phase IVB (Table 2). All of the three children who achieved three consecutive unprompted successful responses in phase V achieved at least 50% accuracy in phase V (Table 2).

**Table 2:**
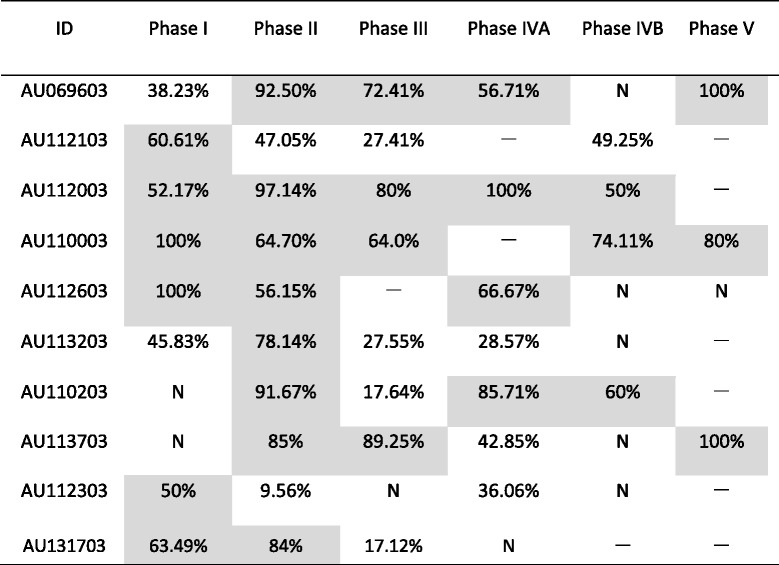
Accuracy rates after three consecutive unprompted successful responses

Gray shading indicates that the trained children achieved at least 50% accuracy in these phases. –: The child was not offered training in this phase; *N*: The child did not achieve three consecutive unprompted successful responses in this phase

Seven children achieved at least 50% accuracy in at least two of the five phases (Table 2 and Additional file 2: Figure S1). AU112103, AU112303, and AU113203 achieved at least 50% accuracy in only one phase (Table 2 and Additional file 2: Figure S1). AU112103 and AU112303 achieved at least 50% accuracy in phase I. AU113203 achieved at least 50% accuracy in phase II. Two children (AU112003 and AU110003) achieved at least 50% accuracy in all of the phases in which they were trained (Table 2 and Additional file 2: Figure S1). AU69603 and AU112603 achieved at least 50% accuracy in phase IVA but not in phase IVB (Table 2 and Additional file 2: Figure S1).

## Discussion

To address the lack of AAC mobile Apps for minimally verbal children with ASD in Mainland China, we developed a flexible and customizable App named Yuudee that runs on mobile touchscreen devices. We evaluated the training effectiveness of making requests with Yuudee in ten minimally verbal children with ASD in Mainland China. All of the children improved in their ability to make requests with Yuudee during training. These ten children achieved three consecutive unprompted successful responses in at least three of the five training phases and at least 50% accuracy in at least one of the five training phases. The design of the training phases was strongly influenced by typical PECS phases and sought to introduce the various functions of Yuudee. The training procedures used in this study improved the requesting skills of minimally verbal children with ASD.

Both single-step and multiple-step requesting skills were targeted in this study. Phases I and II aimed to enable children to touch the pictures on the screen to request favorable items; a single-step process to make a request. Phases III and V addressed the advanced functions of Yuudee. As the distances between the child, the device, and the communication partner increased in phase IV, the child was required to move to and then take the device. Thus, phases III, IV, and V were multiple-step processes. Four children achieved at least 50% accuracy in both phases I and II. Four children achieved at least 50% accuracy in at least two of the three phases from phase III to phase V. Therefore, our multi-session training process for Yuudee improved both single-step and multiple-step requesting skills of minimally verbal children with ASD.

For the trained children who did not achieve three consecutive unprompted successful responses in phases III, IV, and V, the numbers of trials conducted with them were close to or more than the average number of trials conducted with the children who achieved three consecutive unprompted successful responses in these phases. We thus conclude that these phases were difficult for them. For children who obtained a low accuracy after the achievement of three consecutive unprompted successful responses, they usually needed prompts to initiate or correctly complete the requests in many trials. Therefore, the accuracy rates in a sense reflected the speed at which they were able to master these particular phases. The numbers of phases in which they reached at least 50% accuracy appears to reflect the training effectiveness of the training process.

Recall that we used the achievement of 50% accuracy as an evaluation criterion in this study. In phase II, discrimination was initially made between one preferred item and one non-preferred/unrelated item. For all ten of the children, at least six unprompted successful requests were made during eight consecutive trials. This suggested that pictures were not chosen by chance when there were only two pictures on the screen.

Two of the children (AU112103 and AU110003) were not offered training for phase IVA but were directly trained for phase IVB. This was an oversight of the trainers. AU112103 and AU110003 both achieved three consecutive unprompted successful responses and achieved 49.25 and 74.11% accuracy, respectively, in phase IVB. Among the eight other children (i.e., those who were initially trained for phase IVA), four children achieved at least 50% accuracy in phase IVA, and two children achieved at least 50% accuracy in phase IVB. Therefore, it seems that directly offering training for phase IVB was also effective in increasing the spontaneity of the children.

The present study had several limitations. First, there were no follow-up sessions. Although all ten of these children made progress in requesting items during the training, it would be valuable to measure the maintenance of their requesting skills through follow-up sessions in future studies. Second, the training sessions and evaluations were conducted in a classroom. It would be important in future studies to evaluate how their requesting behaviors may be generalized outside of the classroom. Third, in the present study, we did not collect data on whether and how much the parents trained their children to use Yuudee at home between the official training sessions. Training conducted by the parents at home, if it existed, may have affected the training effectiveness that we evaluated. Fourth, there was no control group in the present study, which limited the possible analyses. In future studies, we will add a control group of children who will undertake the same behavior intervention but with no training with Yuudee or training with other AACs. Finally, in addition to the above, more subjects are needed to associate training effectiveness with pre-training factors. Training effectiveness varied considerably between these ten children. It is worth noting that AU112003, who had the lowest expressive age equivalent score among these children, obtained relatively better training effectiveness. AU112003 achieved three consecutive unprompted successful responses and at least 50% accuracy in all of the phases (phases I–IV) in which he was trained. Neither standard scores nor age equivalent scores for the four domains of the Vineland Adaptive Behavior Scales-Chinese version were significantly correlated with the number of training phases for which the children were able to achieve at least 50% accuracy (*p* value > 0.1, Pearson’s correlation). This may relate to the small sample size. It is conceivable that conducting a combined analysis of additional phenotypic factors with the training data collected from more children could help to identify any strong influences related to the impressive performance of children like AU112003. The identification of pre-training characteristics that are associated with training effectiveness could enable us to predict which children may respond particularly well to Yuudee training.

The feature of turning off the speech outputs was added to Yuudee based on the advice of experienced trainers who teach children with autism who have limited functional speech. Testing whether or not turning off the speech outputs could promote the spontaneous speech of children was not one of the primary aims of this study. However, we did make some interesting observations about how three particular children behaved when the speech outputs were turned off in a few trials. AU110003 and AU110203 spoke the name of the reinforcers when they found that no speech was broadcast after they touched a picture. In two trials, AU110003 said the name of another item that he was not asking for after he touched the target picture with no speech output. AU069603 touched the picture of another reinforcer when no speech was broadcast. During training, AU110003 and AU110203 often echoed the phrases outputted by the iPads but AU069603 seldom did.

Each child was assessed using the aberrant behavior checklist (Chinese version) before and after Yuudee training [40]. These children obtained significantly improved scores on the lethargy/social withdrawal subscale after Yuudee training (*p* value = 0.04, one-sided Wilcoxon rank sum test). However, because of the lack of a control group, we were not able to ascertain whether the improvements were the result of Yuudee training as these ten children also attended ABA (applied behavior analysis) training during the 5-week period when they underwent training for making requests with Yuudee.

The parents of some children who were not among the ten trained children in the main study trained their child (with limited spoken language) to make requests with Yuudee at home under the direction of trainers involved in the present study. A 5-year-old boy with ASD who had little spoken language was trained to use Yuudee by his mother at home. The mother reported an event that made her realize that Yuudee could help her son to communicate. One day, the boy cried continually even though she had given him many of his preferred toys. She suddenly thought of Yuudee. After she gave the iPad to the boy, the boy touched the picture “I want the playdough.” When she gave him the playdough the boy calmed down. As reported by the mother, her child could, after a period of sustained training, spontaneously find and use the iPad to make requests at home.

Yuudee is the first AAC mobile App in Mainland China, and in this study, we demonstrated the effectiveness of Yuudee at improving the requesting skills of minimally verbal children with ASD. Because of the widespread use of mobile touchscreen devices, Yuudee is easily available and accessible. Yuudee can be adapted to a wide variety of situations in which children have communication needs. It is conceivable that its use could be included as a natural part of daily life. Considering the free availability of Yuudee and the large number of minimally verbal individuals with ASD in Mainland China and the large global population of Chinese speakers, Yuudee could (in theory) benefit a great many children with ASD and their families. Given what has been reported in studies using other SGDs, it is possible that the sustained use of Yuudee may be helpful for improving spoken language or other functional communication skills (e.g., commenting skills) in children with ASD. Moreover, Yuudee may also be helpful for minimally verbal children with other disabilities.

## Conclusions

We developed an AAC mobile App named Yuudee for minimally verbal children with ASD in Mainland China. The large built-in library of Yuudee has 39 categories of pictures with corresponding spoken phrases that represent the various communication needs of minimally verbal children. Yuudee can be easily customized according to the specific communication needs of the child and the level at which the child operates Yuudee. Yuudee is freely available and runs on iPads and Android phones. The training for making requests with Yuudee consists of five phases that were designed based on the training phases of PECS and the functionalities of Yuudee. All ten of the minimally verbal children with ASD who received Yuudee training achieved three consecutive unprompted successful responses in at least three of the five phases. Three children achieved at least 50% accuracy in only one phase; the other seven children achieved at least 50% accuracy in at least two of the five phases. Two children achieved at least 50% accuracy in all of the phases in which they were trained. Therefore, the use of Yuudee with the training procedures adapted from the PECS training procedures was effective at improving the requesting skills of minimally verbal children with ASD.

## Additional files

Additional file 1: Table S1. The training procedure checklist for phases I–V. (DOCX 16 kb)

Additional file 2: Figure S1. Accuracy rates of each child for the phases in which the child was trained and achieved three consecutive unprompted successful responses. (PDF 951 kb)

## Abbreviations

AAC: Augmentative and alternative communication
ADOS: Autism Diagnostic Observation Schedule
ASD: Autism spectrum disorder
IOA: Inter-observer agreement
PECS: Picture Exchange Communication System
SGDs: Speech-generating devices
