# Supplementary material for: Development and evaluation of a speech-generating AAC mobile app for minimally verbal children with autism spectrum disorder in Mainland China
Source: Mol Autism. 2017 Oct 3;8:52. doi: 10.1186/s13229-017-0165-5 (PMC5627471; doi:10.1186/s13229-017-0165-5)
Supplement: Supplementary file 1 — The training procedure checklist for phases I–V. (DOCX 16 kb) [file 13229_2017_165_MOESM1_ESM.docx]

Table S1. The training procedure checklist for phases I-V

| Phase I | | |
| --- | --- | --- |
| 1 | The reinforcer was placed in the child’s view. | Yes/No/NA |
| 2 | At the beginning of this phase, the communication partner presented the reinforcer to the child for several trials. | Yes/No/NA |
| 3 | The communication partner waited for the child to initiate the request. | Yes/No/NA |
| 4 | If the child reached toward the reinforcer, prompts were provided to guide the child to touch the picture on the screen. | Yes/No/NA |
| 5 | Prompts were provided if the child did not initiate the request. | Yes/No/NA |
| 6 | After the recorded speech was broadcast by the device, the communication partner immediately spoke the name of the reinforcer and gave the reinforcer to the child. | Yes/No/NA |
| 7 | No verbal prompts were given. | Yes/No/NA |
| Phase II | | |
| 1 | The pictures on the screen represented highly preferred and non-preferred/unrelated items. | Yes/No/NA |
| 2 | The communication partner waited for the child to initiate the request. | Yes/No/NA |
| 3 | Prompts were provided if the child did not initiate the request. | Yes/No/NA |
| 4 | If the child touched the target picture, then the communication partner immediately spoke the name of the reinforcer and gave the reinforcer to the child after the recorded speech was broadcast by the device. | Yes/No/NA |
| 5 | If the child touched the picture representing the non-preferred/unrelated item, then the communication partner gave that item to the child after the recorded speech was broadcast by the device. | Yes/No/NA |
| 6 | No verbal prompts were given. | Yes/No/NA |
| Phase III | | |
| 1 | The pictures on the screen page displayed for the child represented non-preferred/unrelated items. | Yes/No/NA |
| 2 | The communication partner waited for the child to initiate the request. | Yes/No/NA |
| 3 | If the child just touched the picture on the screen without switching between different screen pages in the previous trial, then the communication partner provided full physical prompts to guide the child to navigate to the screen page with the preferred item in this trial. | Yes/No/NA |
| 4 | Prompts were provided if the child did not initiate the request. | Yes/No/NA |
| 5 | When the recorded speech was broadcast by the device, the communication partner gave the corresponding item to the child. | Yes/No/NA |
| 6 | No verbal prompts were given. | Yes/No/NA |
| Phase IV | | |
| 1 | There was a distance between the child and the communication partner (Phase IVA), and there were distances between both the child and the communication partner and the child and the device (Phase IVB). | Yes/No/NA |
| 2 | The communication partner waited for the child to initiate the request. | Yes/No/NA |
| 3 | Prompts were provided if the child did not initiate the request. | Yes/No/NA |
| 4 | If the child did not touch the picture in front of the communication partner, prompts were provided to help the child touch the picture in front of the communication partner. | Yes/No/NA |
| 5 | When the recorded speech was broadcast by the device, the communication partner gave the corresponding item to the child. | Yes/No/NA |
| 6 | No verbal prompts were given. | Yes/No/NA |
| Phase V | | |
| 1 | The Yuudee icon was displayed on the screen. | Yes/No/NA |
| 2 | The communication partner waited for the child to initiate the request. | Yes/No/NA |
| 3 | Prompts were provided if the child did not initiate the request. | Yes/No/NA |
| 4 | Physical prompts were provided if the child touched the icon of another application. | Yes/No/NA |
| 5 | When the recorded speech was broadcast by the device, the communication partner gave the corresponding item to the child. | Yes/No/NA |
| 6 | No verbal prompts were given. | Yes/No/NA |
