# Supplementary figures and images for: Development and evaluation of a speech-generating AAC mobile app for minimally verbal children with autism spectrum disorder in Mainland China
Source: Mol Autism. 2017 Oct 3;8:52. doi: 10.1186/s13229-017-0165-5 (PMC5627471; doi:10.1186/s13229-017-0165-5)

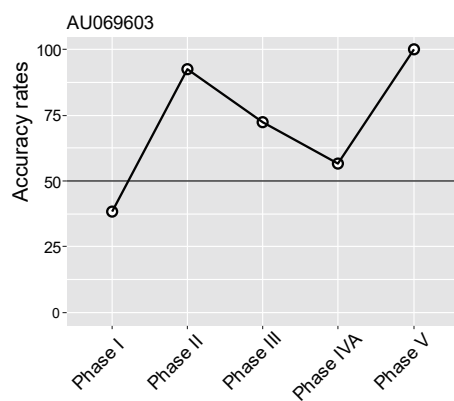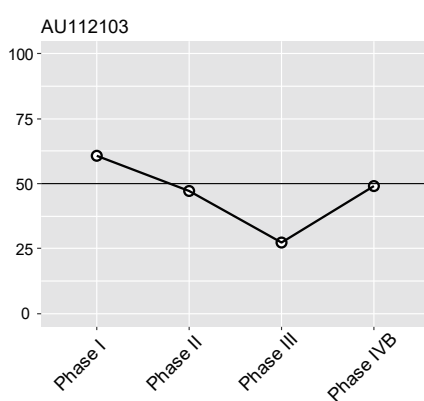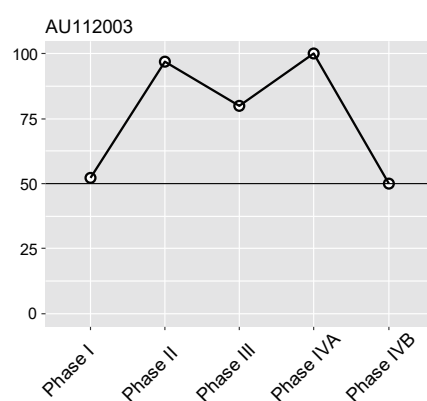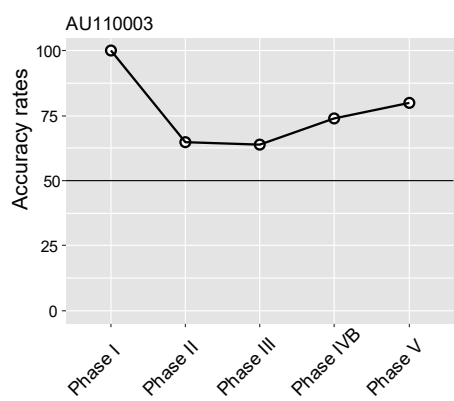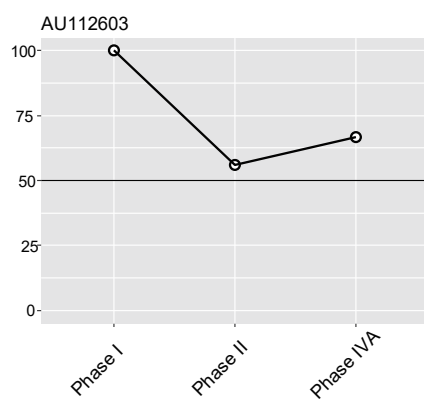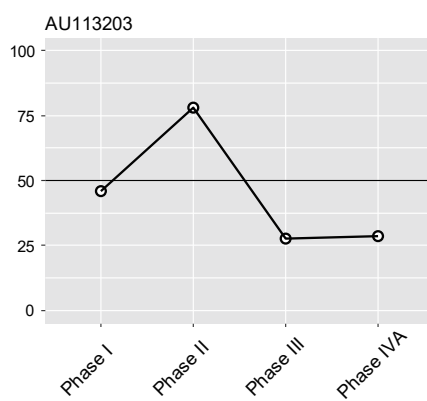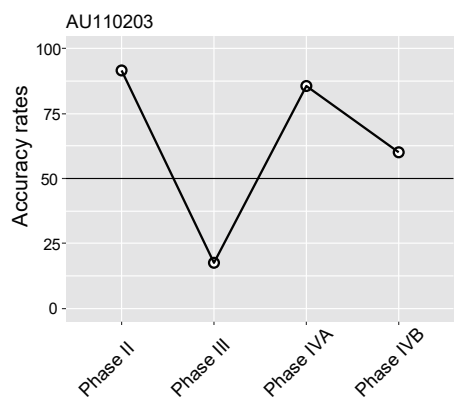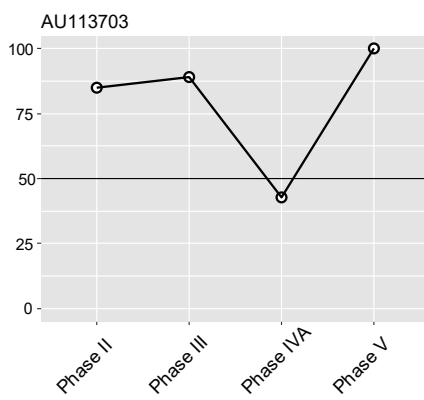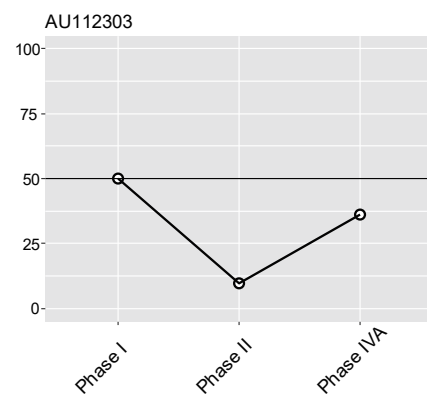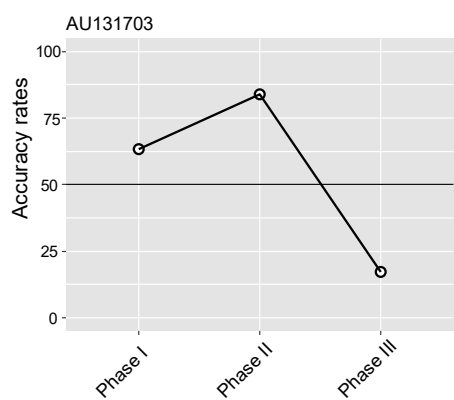

Supplement: Supplementary file 2 — Accuracy rates of each child for the phases in which the child was trained and achieved three consecutive unprompted successful responses. (PDF 951 kb) [file 13229_2017_165_MOESM2_ESM.pdf]
